# Using image mapping towards biomedical and biological data sharing

**DOI:** 10.1186/2047-217X-2-12

**Published:** 2013-09-23

**Authors:** Nurzi Juana Mohd Zaizi, Dayang Nurfatimah Awang Iskandar

**Affiliations:** 1 Department of Computer Science, Heriot-Watt University, Edinburgh, Scotland, EH14 4AS, UK; 2 Faculty of Computer Science and Information Technology, Universiti Malaysia Sarawak, Kota Samarahan, Sarawak, 94300, Malaysia

**Keywords:** Data integration, Spatial relations, Biomedical data, Biomedical image, Image mapping

## Abstract

Image-based data integration in eHealth and life sciences is typically concerned with the method used for anatomical space mapping, needed to retrieve, compare and analyse large volumes of biomedical data. In mapping one image onto another image, a mechanism is used to match and find the corresponding spatial regions which have the same meaning between the source and the matching image. Image-based data integration is useful for integrating data of various information structures. Here we discuss a broad range of issues related to data integration of various information structures, review exemplary work on image representation and mapping, and discuss the challenges that these techniques may bring.

## Review

### Image-based data integration in eHealth domain and life sciences

Biomedical imaging informatics has become a crucial part of modern healthcare, clinical research and basic biomedical sciences. Rapid improvement of imaging technology and advancement of imaging modalities in recent years have resulted in a significant increase in the quantity and quality of such images. Being able to integrate and compare such image-based data has developed into an increasingly critical component in the eHealth domain and life sciences.

Image-based data integration in eHealth and life sciences is typically concerned with the method of anatomical space mapping. Anatomical space mapping involves mapping between spatial regions in the source and matching images in a database. The mapped regions have similar semantics. Image-based data integration is useful for integrating data from various information modalities. For example, patients are now routinely undergoing a variety of digital medical imaging investigations, such as magnetic resonance imaging (MRI) and computed tomography (CT) scanning. The images resulting from these investigations become part of patients’ medical records and are kept indefinitely. The integration of different medical imaging modalities for a single patient can be useful for operations, such as to automatically restaging a condition by comparing the scan from today against the one taken from the previous years, or to predict disease progression. Likewise, the integration of medical imaging modalities from multiple patients with the same disease can yield useful information for diagnosis and prediction; for example, to make automated stratification of patients into different risk categories, or to compare the range of abnormalities in patients. Being able to integrate and compare such image-based data has developed into an increasingly critical component in the life sciences and eHealth domain. It demonstrates potential clinical benefits to retrieve, compare and analyse large volumes of biomedical data for epidemiological studies, educational uses, and monitoring the clinical progress of a patient or translational science purposes.

A biomedical atlas consists of a graphical model, the ontology associated with the graphical model and a mapping between those two. The ontology contains a collection of anatomical domains and relations between those domains. The graphical model is a digital image of an object (e.g., of a human or animal body) along with the identified anatomical domains. Image-based data integration is needed for integrating images and natural-language descriptions in a spatial space. Images may come from biomedical atlases and patients’ clinical images. On the other hand, the natural-language descriptions may come from free text of biomedical literature, radiological reports and other related medical reports. Integrating data between images of biomedical atlases and natural-language descriptions of space from biomedical literature are indeed vital for full and complete results for a gene expression query. Moreover, integrating data between patients’ clinical images and medical reports can be useful for operations such as to search for similar medical cases for diagnosis [1], to systematically evaluate results from clinical images which is necessary to correlate them with the expert judgments of radiologists and other clinical specialists interpreting the images [2], and many more. To enable automated comparison feasible, it is necessary to integrate the knowledge content of the clinical images with the descriptions contained in medical reports.

This paper discusses related work on image representation and mappings. In particular, it focuses on ontology-based and image processing-based techniques. An ontology-based technique represents an image using spatial relations, and mapping can be performed based on the similarity of spatial relationships. Image processing-based technique represents an image using voxel or pixel, and mapping can be performed based on fiducial points.

### Image mapping approaches

In this section two approaches of mapping is presented. The purpose of mapping is to enable anatomical space integration. The discussion is focused on ontology-based mappings using spatial relations and image processing-based mappings using fiducial points.

#### Spatial relations: ontology-based mappings

The first step in mapping based on ontology is to segment the image according to its anatomical regions. Then, the regions are linked to the appropriate concepts in the atlas’ anatomy ontology. Regions from two different images are then mapped according to the similarity of their spatial relationships. For example, if region *a*1 has the relationships *a*1 is adjacent to *b*1, and *a*1 is adjacent to *c*1 then its equivalent region, *a*2, must be adjacent to *b*2 and *c*2. The integration of anatomical space can then be achieved by linking between their respective anatomy ontologies.

The concepts of spatial relations have been well employed in ontologies by both FMA (Foundational Model of Anatomy) [3] and Bittner [4] to describe anatomical space in a biomedical domain. In general, spatial relations between anatomical entities are described using relationships from the following categories: **Mereological relations** describe the concept of parthood between the whole and its parts, for example, finger is *part of* hand, hand is *part of* the arm and so forth. **Topological relations** describe the concept of connectedness among entities, for example, two entities are externally connected if the distance between them is zero and do not overlap; one example is in human major parts of the joint, where the synovial cavity is externally connected to the synovial membrane [4]. **Location relations** describe the concept of relative location between entities that may coincide wholly or partially without being *part of* one another, for example, the brain is located in (but not *part of*) cranial cavity.

A heavily used spatial relation ontology is the OBO (Open Biomedical Ontologies) Foundry which includes various life science disciplines, such as anatomy, health, biochemistry or phenotype [5]. OBO enables the sharing of controlled vocabularies across different biological and medical domains. OBO consists of the Relations Ontology (RO), which model the types of relationships between entities. The Relations Ontology (RO) distinguishes relations between the types of entities. Relations *is_a* and *part_of* are used to model foundational relations. Relations *located_in*, *contained_in* and *adjacent_to* are used to model connecting entities in terms of relations between the spatial regions they occupy. Temporal relations such as *transformation_of*, *derives_from* and *preceded_by* are used to model connecting entities, existing at different times. Participation relations such as *has_participant* and *has_agent* are used to model connecting processes to their bearers.

**Figure 1:**
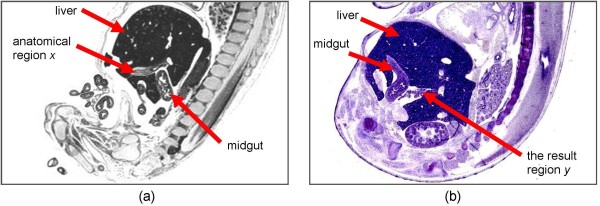
**Spatial Region Mappings.** Based on spatial adjacency between **(a)** anatomical region *x* with other anatomical regions will map *x* to **(b)** the result region *y*.

Topological relations can describe anatomical space in the biomedical domains, particularly on adjacency, discreteness, and connectedness relations. Two entities are described as being adjacent when they are close to each other, but not connected. Discrete entities are not connected. If two entities have a common anatomical space, such that they partially coincide or are externally attached with one another, they are said to be connected. Relations *located_in*, *contained_in* and *adjacent_to* as defined in the Relations Ontology (RO), can describe the location of an anatomical entity in space with respect to other entities. Such relations allow conceptualisation of anatomical space to facilitate the mapping of corresponding regions across images. For example, anatomical region *x* in Figure 1(a) is mapped to the result region *y* in Figure 1(b) if *x* is described as *‘adjacent(x, midgut), adjacent(x, liver)’*[6]. Other commonly used ontologies include RadLex and SNOMED-CT. RadLex describe mereological relationships using relations such as *partOf*, *memberOf* and *continuousWith*, while SNOMED-CT provide basic support for anatomic-specific descriptors by using terms such as *apical, left lower segment, endobrochial* and *panlobular*[7]. Anatomical directional terms such as *left lower segment* (as used in SNOMED-CT) can be used to describe the locations of structures.

Nevertheless, images often have ambiguous regions. These regions could be isolated or disconnected to the rest of the image. The limitation of topological relations (i.e., *located_in*, *contained_in*) as used in the Relation Ontology (RO) is that these relations cannot be used to model the relative position among ambiguous anatomical regions. Relation *adjacent_to* can model the adjacency between two anatomical regions that are located very close to one another. However, for anatomical regions that are isolated or disconnected, such that the relative position involving these regions cannot be described as adjacent because of the distance constraints needs more investigation. Perhaps another approach is to automatically calculate the anatomical spatial location using the approach proposed by [8].

#### Fiducial points: image processing-based mappings

A fiducial point is a point in space, in either 2D or 3D, typically an anatomical landmark that is easily recognizable in an image, usually identified by human experts and possibly assisted by automatic or semi-automated image processing algorithms [6]. Image processing algorithms in [9,10] examine the pixels in an image and classify them into regions. Classification is done based on the pixel’s intensity level. Subsequently, a registration algorithm is used to identify equivalent regions, across images, based on the pixel’s intensity level. Similarly, points of interest (also called fiducial points) are located based on the pixel classification. These fiducial points are typically located at the contours or points of high curvature of objects, for example at tip of the lung and corners of the eyes.

Izard and Jedynak [11] describe a registration approach which employs a Bayesian model to detect these points in order to map between regions across images. Registration technique as proposed by Khaissidi *et al.*[12] use the Hough Transform algorithm to align medical images, based on points of interest extracted from the two compared images. Guest *et al.*[13] use a Gaussian based algorithm to achieve a similar outcome.

### Mapping primitives

This section discusses two types of mapping primitives using spatial relations and fiducial points. Ontology-based mappings may use spatial relations, whilst image processing-based mappings may use fiducial points. These two types of mapping primitives are able to determine corresponding anatomical regions across images.

#### Spatial relations as mapping primitives

Spatial relations describe the spatial relationships between spatial entities. The term ‘spatial’ refers to the location in anatomical space occupied by the anatomical entity. The term ‘entity’ refers to the individual anatomical structure such as liver, heart and kidney. Spatial entities can either be material or immaterial. Material anatomical entities are here understood as anatomical structures with positive mass, such as liver and brain, whereas, immaterial anatomical entities are those anatomical structures with no mass, such as the cavity of the stomach [14]. This comparative study aims to identify existing spatial relations to conceptualise spatial entities in an image. Future research is needed to determine the best set of spatial relations necessary to conceptualise anatomical space of an image to guide the mapping process.

Spatial entities share spatial relationships. Spatial relationships include topological, directional and metric relations [15,16]. These relations can be defined by specifying conditions between entities, such as the distance or the relative position. Topological relations describe topological properties such as connectivity, disjointness and containment between spatial regions. Here, spatial regions are assumed to be parts of an independent background space in which all individuals are located. Eight basic topological relations between two spatial regions according to Egenhofer and Herring [17] are *disjoint, externallyConnected, overlap, contains, equal, coveredBy, inside,* and *covers*.

Metric relations describe the value of the quantitative distance between two spatial entities. Distance can be measured, and it specifies how far is the entity away from the reference entity. Based on distance, relation by means of preposition near or far, as well as adjacency relation, can be defined. For example, near can be defined when the spatial regions, suitably enlarged, have a nonempty intersection. Each spatial region’s width can be enlarged by a fraction of its own height, and vice versa. According to Abella and Kender [18], based on human psychology studies, the value of this fraction is approximately 0.6, particularly, in the case, for long narrow, parallel entities. The relation far, on the other hand, is not the complement of relation near [18]. Far can be defined when the distance between the two enlarged spatial regions *x* and *y*, in either *x* or *y* extent, is larger than the maximum dimension of the two spatial regions in that same *x* or *y* extent. The adjacency relation can be defined between two material anatomical entities that are close, but are not connected. More precisely, the distance between them is small, but non-zero positive distance apart [4].

Directional relations are usually described between two spatial entities that do not overlap [19]. Approximation for these relations can be done by comparing entities representative points (also called centroid) or their minimum bounding boxes. These relations are often described based on cardinal directions between two spatial entities [20]. Work by Frank [21]; Freksa [22]; Ligozat [23] use centroid of spatial entities to define directional relations between two entities. Papadias and Sellis [24] represent each spatial entity using two coordinate points corresponding lower-left and upper-right corner of the entity’s minimum bounding box. Defining directional relations depend on a frame of reference. A frame of reference can be established by assigning a 2D coordinate system to the centroid of spatial entity. The x-axis can then be defined as the west-east axis of the entity. The negative region represents the west of the entity while the positive region represents its east. Doing the same with the y-axis to describe the north and south of the entity, it is then possible to determine directional relations for every spatial entity corresponding to the spatial entity that has the frame of reference. The frame of reference guarantees directional relations between two spatial entities remain the same regardless of their viewpoint. Topological relations are invariant under continuous transformation, such as translation, rotation, or scaling. Directional relations are also invariant under such transformation as a frame of reference can be established [16]. Two spatial entities with a metric distance measure could also change upon scaling but preserve under translation and rotation. Since spatial relations are invariant under continuous transformation, their persistence is fundamental in the process of recognition of anatomical regions in images.

Many existing approaches of image mapping rely on spatial relations between entities of an image. Spatial entities are identified together with spatial relationships among them to represent the image. Mechouche *et al.*[25] present a method to describe spatial relations between sulci and gyri of the brain cortical structure by using the following terms: *anteriorTo, posteriorTo, superiorTo, inferiorTo, lateralTo* and *medialTo*. Hudelot *et al.*[26] present a method to compute the implementation of spatial relations terms such as *right_Of, left_Of, close_to, very_close_to, external boundary* and *internal boundary* to describe the brain cerebral. Du *et al.*[27] present a method which involves topological and directional relations to define some natural language spatial relations. They propose the following directional natural-language terms: *EP* to denote natural language *east part of a region*, *WP* to denote natural language *west part of a region*, *SP* to denote natural language *south part of a region* and *NP* to denote natural language *north part of a region*. These work demonstrate that the recognition of spatial entities depends on entities’ spatial relationships in an image.

Chang and Wu [28] propose a technique called 9DLT matrix which apply nine directional codes to represent spatial relationships. They define directional code as follows: 0 to denote *east*, 1 to denote *northeast*, 2 to denote *north*, 3 to denote *northwest*, 4 to denote *west*, 5 to denote *southwest*, 6 to denote *south*, 7 to denote *southeast*, and 8 to denote *equal*. A single triple *(x, y, r)*, denotes a spatial relation between two spatial entities *x* and *y*. Directional code *r*=0 represents *y* is to the east of *x*, for instance. Subsequently, a set of triples represents an image. Two images are then mapped according to the similarity of their spatial relationships based on the corresponding set of triples. However, the 9DLT matrix has a significant drawback under rotation of direction. Mapping between two identical images, where the first image is 90 degrees rotated version of the second image, though these two images represent the same image, according to 9DLT matrix, these two images do not match as their corresponding sets of triples are totally different due to 90 degrees rotation of direction.

Guru and Punitha [29] propose to address the limitation of 9DLT matrix by modelling directional relations between two spatial entities using a directed line segment. A directed line segment is a line joining between two distinct entities. For example, the line joining the entity *x* to entity *y* becomes the line of reference, and the corresponding direction from entity *x* to entity *y* becomes the direction of reference for the image. The approach computes the direction of the line joining *x* to *y* using Euclidean distance prior to obtain the direction of reference. The relative pair-wise spatial relationships between each pair of entities are perceived with respect to the direction of the line of reference. In order to make the system invariant to image transformations, the direction of reference is conceptually aligned with that of the positive x-axis of the coordinate system. The proposed improvement method by Guru and Punitha [29], successfully overcome the deficiency in 9DLT matrix, however, the method only cover directional information, which means information on topology is lost.

Karouia and Zagrouba [30] propose to represent spatial relationships between two spatial entities of an image using entity relative positioning vector. The set of these vectors provides information about the disposition of different entities of the image. The approach defines this disposition based on five component vectors. These vectors are positioning degree on the left, on the right, on top, below and of inclusion. Each of these elements express a degree of positioning by a numeric value between 0 and 1. This method is intended to represent images containing only isolated entities. Hence, information on topology is not required, as to why the approach does not contain any concept on connectedness among spatial entities.

Zhou *et al.*[31] propose a method called Augmented Orientation Spatial Relationship (also called as AOSR) to describe a range of directions between two spatial entities of an image. Assume that two images *c*1 and *c*2 both have the same entities *x* and *y*; however, the relative distance between these entities in both images is different. If one simply says for image *c*1, entity *x* is at the northeast of entity *y* (according to the centroid of *x* and *y*), then there is no difference between entities *x* and *y* in image *c*2. Therefore, the focus of AOSR is to capture relative distance between spatial entities prior to describe directional relations between them. Though topological information is also not covered in AOSR, Zhou *et al.*[31] claim that the approach may simply be combined with Egenhofers topological representation, to cover for topological information.

Kulkarni and Joshi [32] and Majumdar *et al.*[33] propose a method, which combines both topological and directional relations. However, the method does not capture the notion of distance between spatial entities, such that there is no difference between two entities that are quite near or far to one another.

Wang [34] proposes a method by the use of spatial operator *Σ* to capture interval between the minimum bounding boxes of two spatial entities. This method apparently removes precise spatial description, between entities. The operator indicates there is a space between the two entities that could be either disjoint, near or far. Given a description like *ΣfemurΣmetanephrosΣ*, it led to spatial knowledge that *femur* and *metanephros* are disjoint, but it led to uncertainty as to whether these two spatial entities are near or are they far to one another.

Yang and Zhongjian [35] propose an image representation structure using the Mixed Graph Structure (MGS). They demonstrate their method on medical images. The method first extracts spatial entities as primitives. These spatial entities are then organised into a mixed graph structure according to their spatial relations. The approach uses only two types of spatial relations, which are *inclusion* and *adjacency*.

Overall, most image description and mapping approaches in [29,31,34] use spatial relations of entities in an image. Methods in [32,33] account on both topological and directional relations of spatial entities. Approaches in [30,33,35] represent images as graphs. The graphs conceptualise spatial relations between entities and then solve the mapping as graph matching problem.

#### Fiducial points as mapping primitives

Some image processing-based mappings use fiducial points as the mapping primitive, where these algorithms use a set of fiducial points to determine corresponding anatomical regions between images. Fiducial points are anatomical landmarks in the anatomy that experts use to determine biologically meaningful correspondence between structures [36]. Two images are then aligned to one another by knowing pairs of corresponding fiducial points in each image. These fiducial points are typically located at the contours of the images or points of high curvature like corners of objects, for instance. Because there is currently no standardized set of fiducial points, this comparative study aims to identify examples of fiducial points that have been detected. Further research is needed to determine the best combination of fiducial points necessary to conceptualise anatomical space of an image to guide the mapping process. Getting high accuracy with a large number of fiducial points is not the goal.

Georgescu *et al.*[37] propose a machine learning method to detect fiducial points on a large set of ultrasound heart images in medical databases. These heart images have large variation in appearance and shape. Detection of fiducial points and anatomical regions involved a two-step learning problem – structure detection and shape inference.

Potesil *et al.*[38] and Seifert *et al.*[39] provide recent examples on research work involving segmentation of fiducial points and the corresponding anatomical regions. Potesil *et al.*[38] proposed a method to detect 22 fiducial points based on dense matching of parts-based graphical models. These fiducial points are C2 vertebra, C7 vertebra, top of the sternum, top right lung, top left lung, aortic arch, carina, lowest point of sternum (ribs), lowest point of sternum (tip), Th12 vertebra, top right kidney, bottom right kidney, top left kidney, bottom left kidney, L5 vertebra, right spina iliaca anterior superior, left spina iliaca anterior superior, right head of femur, left head of femur, symphysis, os coccygeum, and center of bladder.

Seifert *et al.*[39] proposed a method for the localization of 19 fiducial points for whole-body scan. These fiducial points are left and right lung tips, left and right humerus heads, bronchial bifurcation, left and right shoulder blade tips, inner left and right clavicle tips, sternum tip bottom, aortic arch, left and right endpoints of rib 11, bottom front and back of the L5 vertebra, coccyx, pubic symphysis top and the left and right front corners of the hip bone. They also have trained ten anatomical region centers – four heart chambers, liver, kidneys, spleen, prostate and bladder.

These fiducial points are useful to estimate anatomical regions that are present, as well as their most probable locations and boundaries in an image [39]. Subsequently, these fiducial points can be used to establish reliable correspondences between anatomical regions across different images.

### Discussion

The integration of biomedical images between biomedical atlases is needed to enable these data sources not only to share information, but to allow a user to use information from all related resources. For example, the mouse embryo gene expression data may come from different data sources. Many of these data sources which are, in general, disconnected from each other making it difficult to see the overall results of a particular experiment. The Allen Developing Mouse Brain Atlas is a data source storing gene expression data across seven developmental stages of the mouse brain [40]. EMAGE [41] is another example of mouse atlas covering gene expression data for anatomical structures corresponding EMAP Anatomy Ontology [42]. Gene expression data for the mouse brain is also available from EMAGE. Another example of a mouse atlas that provides gene expression data for the mouse brain is the GENSAT brain atlas. GENSAT is a gene expression atlas of both the developing and adult mouse, and stores gene expression data for anatomical structures corresponding brain and spinal cord [43]. Due to different experimental designs and various analysis of results, data in these online resources can be different and inconsistent [44]. In addition, different update routines can cause data from these atlases to be incomplete. The consequence is these atlases may provide different result even for the same gene expression query. To illustrate this, consider the gene *Efna2* and the structure of midbrain at Theiler Stage 19. At the time of writing this paper the EMAGE contains two experiments for this combination, and it suggests that *Efna2* is expressed. The Allen Developing Mouse Brain Atlas also has this structure at the same developmental stage and indicate that *Efna2* is also expressed. GENSAT brain atlas also has this structure, however, indicate that there are currently no experimental results in their database for gene *Efna2*. With available evidence from EMAGE and The Allen Developing Mouse Brain Atlas, the most likely conclusion is that the gene *Efna2* is expressed in midbrain at TS19, however if the user depends on a single resource, in this case the GENSAT brain atlas, a wrong conclusion may be drawn. Because the data from these resources are sometimes incomplete, it is vital that all resources are used to generate a full and complete query results [45].

This paper proposes to achieve this integration by mapping the images of biomedical atlases. However, the implementation of this approach involves a number of problems. First, different biomedical atlases may have a different number of segmented regions in their images, causing one structure to correspond to parts of several structures, and vice versa. The mapping of images in order to achieve biomedical atlases integration may require alignment representations of anatomy differing in structure and domain coverage. Second, these images may have the exact same anatomical structures but the morphology may vary with scale, orientation and the position of the structure. Third, different biomedical atlases may have the same segmented images, but may use different anatomical names causing interoperability issue to find corresponding anatomical regions between these images. An efficient representation structure is necessary to conceptualise anatomical space of an image to guide the mapping process. It is hoped that by having a mechanism to describe anatomical space using fiducial points and a set of spatial relations can guide the mapping of images across biomedical atlases to facilitate the integration of these data sources. It is a middle approach that could be attempted when the image processing-based solution is unavailable, or when the ontology-based solution has difficulties.

## Conclusions

This paper provides an overview of work on image representation and mapping by exploring concepts of spatial relations within the ontology-based approach, and examples of fiducial points within the image processing approach. The contribution of this paper is in identifying the first step towards using image-based data integration for integrating biomedical atlases via image-based data integration. Of the existing solutions to image mapping, ontology-based methods often lack spatial precision. Image processing methods have difficulties when the underlying morphologies are too different. An efficient representation structure is necessary to conceptualise anatomical space of an image to guide the mapping process. The question is; what is the best set of spatial relations to describe a biomedical domain? Additionally, which anatomical landmark should be selected as fiducial points to provide good spatial precision? Most importantly, a vigorous effort is needed to investigate how to perform mapping without using a large concept of spatial relations nor using a huge number of fiducial points. However, this work covers a specific domain, which is the mapping between images of biomedical atlases. Further research is needed to facilitate data integration between biomedical atlases with other resources, such as natural-language description of space (i.e., radiological report and biomedical literature) [46,47] and database warehouses (i.e., structured database of biomedical facts) [48-51], which could heavily involved knowledge representation systems such as OWL (Web Ontology Language) and RDF (Resource Description Framework).

## Abbreviations

AOSR: Augmented orientation spatial relationship; EMAP: Edinburgh mouse atlas project; EMAGE: e-Mouse atlas of gene expression; GENSAT: Gene expression nervous system atlas; RadLex: Radiology lexicon; RO: Relations ontology; SNOMED-CT: Systematized nomenclature of medicine-clinical terms; OBO: Open biomedical ontologies.

## Competing interests

The authors declare that they have no competing interests.

## Authors’ contributions

NJMZ carried out the research work related to fiducial points and its write up in this paper. DNFAI research is related to spatio-temporal relationships in biomedical atlases and assisted in writing this paper. Both authors read and approved the final manuscript.

## References

[B1] Haux R Ammenwerth E Herzog W Knaup P Health care in the information society. A prognosis for the year 2013 Int J Med Inform 2002 66 3 21 10.1016/S1386-5056(02)00030-8 12453552

[B2] Kulikowski CA Gong L Mezrich RS Knowledge-based medical image analysis and representation for integrating content definition with the radiological report Methods Inf Med 1995 34 96 103 9082144

[B3] Rosse C Mejino JLV Burger A, Davidson D, Baldock R The foundational model of anatomy ontology Anatomy Ontologies for Bioinformatics: Principles and Practise 2008 London: Springer-Verlag 59 117

[B4] Bittner T Logical properties of foundational mereogeometrical relations in bio-ontologies Appl Ontology 2009 4 2 109 138

[B5] Smith B Ashburner M Rosse C Bard J Bug W Ceusters W Goldberg L Eilbeck K Ireland A Mungall C Consortium O Leontis N Rocca-Serra P Ruttenberg A Sansone S Scheuermann R Shah N Whetzel P Lewis S The OBO Foundry: coordinated evolution of ontologies to support biomedical data integration Nat Biotechnol 2007 25 11 1251 1255 10.1038/nbt1346 17989687 PMC2814061

[B6] Zaizi NJM Burger A Kostkova P, Szomszor M, Fowler D Towards spatial description-based integration of biomedical atlases4th ICST International Conference on eHealth (eHealth 2011): 21-23 November; Malaga, Spain 2012 Berlin, Heidelberg: Springer-Verlag 196 203

[B7] Alex AB Ricky KT Medical Imaging Informatics 2010 New York: Springer

[B8] Iskandar D Visual ontology query language1st International Conference on Networked Digital Technologies (NDT ‘09) 2009 65 70

[B9] Boccignone G Napoletano P Ferraro M Embedding diffusion in variational bayes: A technique for segmenting images Int J Pattern Recognit Artif Intell World Sci 2008 22 811 827 10.1142/S0218001408006533

[B10] Wyawahare MV Patil PM Abhyankar HK Image registration techniques: an overview J Image Process Pattern Recognit 2009 2 3 11 28

[B11] Izard C Jedynak B Bayesian registration for anatomical landmark detection Proceedings of 3rd IEEE International Symposium on Biomedical Imaging 2006 856 859

[B12] Khaissidi G Tairi H Aarab A A fast medical image registration using feature points ICGST-GVIP J 2009 9 3 19 24

[B13] Guest E Berry E Baldock RA Fidrich M Smith MA Robust point corespondence applied to two and three dimensional image registration IEEE Trans Pattern Anal Mach Intell 2001 23 2 1 15

[B14] Bittner T Donelly M Goldberg LJ Neuhaus F Burger A, Davidson D, Baldock R Modeling principles and methodologies - spatial representation and reasoning Anatomy Ontologies for Bioinformatics: Principles and Practise 2008 London: Springer-Verlag 307 326

[B15] Li S Combining topological and directional information for spatial reasoning Proceedings of the 20th International Joint Conference on Artifical Intelligence, IJCAI‘07 2007 San Francisco: Morgan Kaufmann Publishers Inc. 435 440

[B16] Schwering A Evaluation of a semantic similarity measure for natural language spatial relations Proceedings of the 8th International Conference on Spatial Information Theory, COSIT‘07 2007 Berlin, Heidelberg: Springer-Verlag 116 132

[B17] Egenhofer MJ Herring J Categorizing binary topological relations between regions, lines and points in geographic databases Tech 1991 Department of Surveying Engineering, University of Maine

[B18] Abella A Kender JR From images to sentences via spatial relations Proceedings of the Integration of Speech and Image Understanding 1999 117 146

[B19] Liu Y Guo Q Kelly M A framework of region-based spatial relations for non-overlapping features and its application in object based image analysis ISPRS J Photogrammetry Remote Sensing 2008 63 4 461 475 10.1016/j.isprsjprs.2008.01.007

[B20] Chen J Jia H Liu D Zhang C Composing cardinal direction relations basing on interval algebra Proceedings of the 4th International Conference on Knowledge Science, Engineering and Management, KSEM‘10 2010 Berlin, Heidelberg: Springer-Verlag 114 124

[B21] Frank AU Qualitative spatial reasoning: cardinal directions as an example Int J Geogr Inf Sci 1996 10 3 269 290

[B22] Freksa C Using orientation information for qualitative spatial reasoning Proceedings of the International Conference GIS - From Space to Territory: Theories and Methods of Spatio-Temporal Reasoning on Theories and Methods of Spatio-Temporal Reasoning in Geographic Space 1992 London: Springer-Verlag 162 178

[B23] Ligozat G Reasoning about cardinal directions J Vis Lang Comput 1998 9 23 44 10.1006/jvlc.1997.9999

[B24] Papadias D Sellis T Qualitative representation of spatial knowledge in two-dimensional space VLDB J 1994 3 4 479 516 10.1007/BF01231605

[B25] Mechouche A Morandi X Golbreich C Gibaud B A hybrid system for the semantic annotation of Sulco-Gyral anatomy in MRI images Proceedings of the 11th International Conference on Medical Image Computing and Computer-Assisted Intervention - Part I, MICCAI ‘08 2008 Berlin, Heidelberg: Springer-Verlag 807 814 10.1007/978-3-540-85988-8_96 18979820

[B26] Hudelot C Atif J Bloch I Fuzzy spatial relation ontology for image interpretation Fuzzy Sets Syst 2008 159 15 1929 1951 10.1016/j.fss.2008.02.011

[B27] Du S Qin Q Chen D Wang L Spatial data query based on natural language spatial relations Proceedings of the Geoscience and Remote Sensing Symposium (IGARSS ‘05), 2005 1210 1213

[B28] Chang CC Wu TC An exact match retrieval scheme based upon principal component analysis Pattern Recogn Lett 1995 16 5 465 470 10.1016/0167-8655(95)00002-X

[B29] Guru DS Punitha P An invariant scheme for exact match retrieval of symbolic images based upon principal component analysis Pattern Recogn Lett 2004 25 73 86 10.1016/j.patrec.2003.09.003

[B30] Karouia I Zagrouba E New image matching method based on spatial region interrelationships Proceedings of the 4th International Conference on Innovations in Information Technology (IIT ‘07) 2007 675 679

[B31] Zhou XM Ang CH Ling TW Image retrieval based on object’s orientation spatial relationship Pattern Recogn Lett 2001 22 5 469 477 10.1016/S0167-8655(00)00123-9

[B32] Kulkarni MA Joshi RC Content-based image retrieval by spatial similarity Def Sci J 2002 52 3 285 291

[B33] Majumdar AK Bhattacharya I Saha AK An object-oriented fuzzy data model for similarity detection in image databases IEEE Trans Knowl Data Eng 2002 14 5 1186 1189 10.1109/TKDE.2002.1033783

[B34] Wang YH Image indexing and similarity retrieval based on a new spatial relation model 2001 International Conference on Distributed Computing Systems Workshops (ICDCSW ‘01) 2001 396 401

[B35] Yang L Zhongjian T A novel approach for image representation and matching based on mixed graph structure Computational Intelligence and Software Engineering (CiSE 2009) 2009 1 4

[B36] Izard C Jedynak B Stark C Larsen R, Nielsen M, Sporring J Spline-based probabilistic model for anatomical landmark detection Medical Image Computing and Computer-Assisted Intervention (MICCAI 2006), 2006 Berlin, Heidelberg: Springer-Verlag 849 856 10.1007/11866565_104 17354970

[B37] Georgescu B Zhou XS Comaniciu D Gupta A Database-guided segmentation of anatomical structures with complex appearance Proceedings of the 2005 IEEE Computer Society Conference on Computer Vision and Pattern Recognition (CVPR‘05) 2005 Washington: IEEE Computer Society 429 436

[B38] Potesil V Kadir T Platsch G Brady M Improved anatomical landmark localization in medical images using dense matching of graphical models Proceedings of the British Machine Vision Conference 2010 BMVA Press 37.1 37.10

[B39] Seifert S Barbu A Zhou SKevin Liu D Feulner J Huber M Suehling M Cavallaro A Comaniciu D Hierarchical parsing and semantic navigation of full body CT data Proc 2009 725902 725902–8

[B40] Allen Brain Atlas http://developingmouse.brain-map.org

[B41] Christiansen JH Yang Y Venkataraman S Richardson L Stevenson P Burton N Baldock RA Davidson DR EMAGE: a spatial database of gene expression patterns during mouse embryo development Nucleic Acids Res 2010 34 suppl 1 D637—D641 16381949 10.1093/nar/gkj006 PMC1347369

[B42] Baldock RA Bard JB Burger A Burton N Christiansen J Feng G Hill B Houghton D Kaufman M Rao J Sharpe J Ross A Stevenson P Venkataraman S Waterhouse A Yang Y Davidson DR EMAP and EMAGE - a framework for understanding spatially organized data Neuroinformatics 2003 4 309 325 15043218 10.1385/NI:1:4:309

[B43] Gensat Brain Atlas of Gene Expression http://www.gensat.org/index.html

[B44] McLeod K Burger A Towards the use of argumentation in bioinformatics: a gene expression case study Bioinformatics 2008 24 304 312 10.1093/bioinformatics/btn157 PMC2718635 18586728

[B45] Boline J Lee EF Toga AW Digital atlases as a framework for data sharing Front Neurosci 2008 2 100 106 10.3389/neuro.01.012.2008 18982112 PMC2570073

[B46] Yang C Zeng E Li T Narasimhan G Clustering genes using gene expression and text literature data Proceedings of the 2005 IEEE Computational Systems Bioinformatics Conference 2005 Washington: IEEE Computer Society 329 340 10.1109/csb.2005.23 16447990

[B47] Hearst MA Untangling text data mining Proceedings of the 37th Annual Meeting of the Association for Computational Linguistics on Computational Linguistics, ACL ‘99 1999 Stroudsburg: Association for Computational Linguistics 3 10

[B48] Pasquier N Pasquier C Brisson L Collard M Mining gene expression data using domain knowledge Int J Softw Inform 2008 2 2 215 231

[B49] Hemert J Baldock R Hochreiter S, Wagner R Mining spatial gene expression data for association rules Bioinformatics Research and Development, 2007 Berlin, Heidelberg: Springer 66 76

[B50] Schaefer G Nakashima T Data mining of gene expression data by fuzzy and hybrid fuzzy methods IEEE Inf Technol Biomed 2010 14 23 29 10.1109/TITB.2009.2033590 19846381

[B51] Gerner M Nenadic G Bergman CM An exploration of mining gene expression mentions and their anatomical locations from biomedical text Proceedings of the 2010 Workshop on Biomedical Natural Language Processing, BioNLP ‘10 2010 Stroudsburg: Association for Computational Linguistics 72 80

